# Involvement of the anterior insula and frontal operculum during *wh*-question comprehension of *wh*-in-situ Korean language

**DOI:** 10.1371/journal.pone.0298740

**Published:** 2024-04-26

**Authors:** Haeil Park, Jiseon Baik, Hae-Jeong Park

**Affiliations:** 1 Department of English Language and Literature, Kyung Hee University, Seoul, Republic of Korea; 2 Center for Systems and Translational Brain Sciences, Institute of Human Complexity and Systems Science, Yonsei University, Seoul, South Korea; 3 Department of Cognitive Science, Yonsei University, Seoul, Republic of Korea; 4 BK21 PLUS Project for Medical Science, Department of Nuclear Medicine, Department of Psychiatry, Yonsei University College of Medicine, Seoul, South Korea; Tohoku University, JAPAN

## Abstract

In this research, we employed functional magnetic resonance imaging (fMRI) to examine the neurological basis for understanding wh-questions in wh-in-situ languages such as Korean, where wh-elements maintain their original positions instead of moving explicitly within the sentence. Our hypothesis centered on the role of the salience and attention network in comprehending wh-questions in wh-in-situ languages, such as the discernment of wh-elements, the demarcation between interrogative types, and the allocation of cognitive resources towards essential constituents vis-à-vis subordinate elements in order to capture the speaker’s communicative intent. We explored subject and object wh-questions and scrambled wh-questions, contrasting them with yes/no questions in Korean. Increased activation was observed in the left anterior insula and bilateral frontal operculum, irrespective of the wh-position or scrambling of wh-element. These results suggest the interaction between the salience and attentional system and the syntactic linguistic system, particularly the left anterior insula and bilateral frontal operculum, in comprehending wh-questions in wh-in-situ languages.

## 1. Introduction

The goal of the perceiver in a conversation or sentence comprehension is to grasp the speaker’s intention. For the perceiver, a wh-question word in a question sentence elicits attention and helps clarify what the speaker is asking. For example, in the sentence “I wonder whether he bought flowers in the shop,” it is hard to know what the speaker is wondering about. If the speaker says, “I am wondering who bought flowers in the shop,” what the speaker is actually wondering becomes obvious as the wh-interrogative word (“who”) becomes prominent (“salient” in neuroscience terminology) to the perceiver.

In an English-like language, what syntactically distinguishes wh-questions from yes/no questions is the movement of the wh-element to the beginning of the sentence. *Wh*-phrases in languages such as English and Hebrew are argued to overtly move to the front position after applying transformational rules [1]. This movement may provide a pragmatic advantage in clearly pointing out the question of interest while delivering one’s intention as well as capturing others’ intentions. However, in the case of *wh*-in-situ languages such as Korean and Mandarin Chinese, *wh*-phrases in main and embedded questions do not move in overt syntax and stay in their base-generated positions. Nevertheless, it is argued that covert movement takes place at the semantic interpretation or logical form (LF) level in these wh-in-situ languages [2–6].

To enable comprehension of *wh*-phrases in the LF level of the wh-in-situ language, we expected more involvement of complex cognitive and linguistic processes than in overt *wh*-movement languages where the *wh*-element is found at the beginning of a sentence. When faced with a *wh*-element as an interrogative word, a *wh*-element is perceived as salient and captures one’s attention as a pivotal framework for interpreting the whole sentence. The perception of a wh-element as a salient word takes place in the wh-movement languages, but more of the perception is expected in the wh-in-situ languages: the wh-element in the middle of a sentence is less expected, and when detected, it initiates more attention to rapidly reallocating resources for subsequent processing. The wh-sentence comprehension process differentiates two types of question sentences, i.e., yes/no questions and wh-questions. It distinguishes the essential from the subordinate in capturing the speaker’s intention, which is followed by prioritizing the essential in the first place as a contrast with the remaining words. For the perceiver, a wh-question word in a question sentence elicits attention and helps clarify what the speaker is asking.

The *wh*-dependency is another aspect in which wh-questions differ from yes/no questions. In a “wh-movement” language like English, the wh-phrase is moved from its base position that has the original thematic role. A “filler” refers to the displaced wh-element, whereas the original position is called a “gap.” These are said to be dependent on each other to be successfully interpreted. This is because a filler is generally ambiguous with respect to its grammatical function (such as subject or object) and thematic role (agent or patient) until the comprehender encounters the gap position, being able to resolve this ambiguity. Wh-dependencies that do not appear to present a non-local filler-gap relationship on the surface are found in different forms in many wh-in-situ languages, such as Chinese, Japanese, and Korean. These are commonly referred to as wh-in-situ dependencies because the wh-phrase occurs in the location where it is interpreted as in “He sells what?.” There is no visible element in the dependency until the wh-phrase is encountered in its thematically interpreted position. Instead, once the wh-phrase is met, the comprehender must identify its scope relationship with the other sentence operators.

For example, in Korean wh-questions, the wh-phrase stays in its original base position rather than being moved to the beginning of a clause, as shown in (1) below.

John-i mwues-ul mekess -ni?

John-NOM(INATIVE) what-ACC(USATIVE) ate- Q(UESTION)

When forming a wh-question, the wh-element requires a Q-particle like -ni or -nunci on the matrix verb to have an interrogative meaning. Without such a Q-particle, the wh-expression is interpretable as an indefinite pronoun (see Chung 1996, Hong 2005 [7, 8]). In Korean, the position of the Q-particle indicates the interrogative scope to comprehend a sentence (See Appendix 1). The same applies to Japanese, where the use of wh-words typically involves adding a Q-particle, either -ka or -no (meaning ’whether’), at the end of the clause. However, in colloquial Japanese, it is acceptable to form wh-questions without these question particles as long as the appropriate prosody or intonation is used.

To sum up the perceptual processing of wh-questions, the brain differentiates wh-question from yes/no question or general sentences according to an interrogative *wh*-word encountered either in the beginning or middle of the sentence and searches for *wh*-dependency to indicate the scope of the wh-question in both wh-movement or wh-in-situ languages. Since the position of a *wh*-element in a sentence is not predetermined in the wh-in-situ language, the *wh*-element “in situ” encountered in the middle of a sentence may induce salience to initiate searching for Q-particle and specify the essential to grasp the speaker’s intention. In contrast to overt *wh*-movement languages, this process demands more resources because it should be conducted while holding in memory prior parts of sentences that occurred before the *wh*-element during online processing.

Until now, there are very few fMRI studies regarding wh-sentence comprehension compared to yes/no questions in an English-like wh-movement language [9], despite a great deal of neuroimaging studies on relative and cleft sentences. Furthermore, to the best of our knowledge, there are few neuroimaging studies on wh-in-situ languages, except for an event-related potential of electroencephalogram study regarding Japanese Q-particles [10]. Thus, exploring how the brain responds to wh-questions relative to yes/no questions in a wh-in-situ language may not only shed light on neural processing specific to a wh-in-situ language but also help grasp the brain’s syntactic processing in general.

In a functional magnetic resonance imaging (fMRI) study, Ben-Shachar, Palti [9] reported that, in Hebrew, an overt wh-movement language, perception of overt *wh*-movement recruits more the left inferior frontal gyrus (IFG), left ventral precentral sulcus, and bilateral posterior superior temporal sulcus compared to no movement. Since the left IFG is also activated for topicalized sentences, the authors interpreted the IFG activation as a result of syntactic movement. However, what specific process this IFG involvement represents in the syntactic movement during the wh-question comprehension is questionable for an overt wh-movement language where a *wh*-element is given to the listener after being moved to the first place. The syntactic process may be associated with subsequent processes, such as searching for the original position, i.e., ‘gap’ to complete the scope. Exploring the brain processes for *wh*-sentences in a *wh*-in-situ language could help test this prediction. If a similar regional activity exists in a *wh*-in-situ language, the IFG might be associated with syntactic processes common to both language groups.

The current study used fMRI to explore brain responses to *wh*-question sentences in Korean, a *wh*-in-situ language, compared to yes/no questions. To explore the neurocognitive details of the *wh*-sentence processing, we investigated the comprehension of three types of Korean *wh*-questions, i.e., subject and object *wh*-questions and scrambled *wh*-questions, compared to yes/no questions. We further divided wh-questions into two different contrasts: 1) subject vs. object wh-question, 2) scrambled wh-question vs. canonical wh-question.

By contrasting subject and object wh-question constructions in Korean, we tested the effect of the first position for the wh-element. The subject and object wh-questions refer to sentences with the wh-phrases occurring in the position of the subject and the object, respectively, in wh-in-situ languages. The subject wh-element is usually placed in the first position of a sentence, while the object wh-element is not. Korean differs in this respect from English-like overt wh-movement languages, where all wh-elements occur in the first place with high certainty. In the English-like language, object wh-sentences, and relative sentences compared to subject wh-sentences and relatives demand more cognitive resources and elicit more activation in areas including IFG (or Broca’s area) (e.g., Just et al. [60], 1996; Caplan, 2001 [61]). In contrast, both subject and object wh-elements of wh-in-situ languages stay at their canonical positions. The subject wh-element is located at the first place of the sentence in Korean, similarly to the wh-movement languages, while the object wh-element is not. If subject construction activates primary language regions, specifically the IFG, while its object counterpart does not, the first position of wh-element is crucial in inducing neural activation at the IFG at the syntactic level. On the other hand, if there is no significant difference in the IFG activation between these two constructions, the IFG activation in the wh-question comprehension may not be attributable to the first-position-dependent syntactic processes. In the latter case, the IFG activation could instead be due to a shared mechanism between the subject and object construction for comprehending wh-sentences, like dependency processing.

We also explored Korean object scrambling of the *wh*-element. In this syntactic process, the object to be emphasized is extracted from its original position in a verb phrase and moved to a structurally higher place, resulting in a more complex structure. Here, we refer to “object scrambling” simply as “scrambling.” Scrambling is not allowed in English but is permitted in Korean and Japanese. In Korean, a verb-final language, the order of Subject—Object—Verb can be converted, or “scrambled,” to Object—Subject—Verb, without interfering with the grammaticality or truth value of the sentence. For example, “Mary-ga sakwa-lul sass-ta” (Mary-nominative (Nom) apple-accusative (Acc) buy-Past) can be scrambled as “sakwa-lul Mary-ga sass-ta” (apple-Acc Mary-Nom buy-Past). Korean scrambling is semantically vacuous, although initial sentence expressions receive focus for pragmatic reasons. Considering that scrambling can be conducted for the object *wh*-phrase, whether it is scrambled or not should be determined first. This determination is made by checking postpositions or case markers for the subject and object. Indeed, both subject *wh*-element without scrambling and object *wh*-element with the scrambling have *wh*-element in the first place of the sentence. However, scrambling differs from subject or object wh-questions: subject and object wh-questions are canonical in Korean syntax while scrambling is used pragmatically to emphasize the scrambled object.

Despite differences in details, the three *wh*-conditions (i.e., subject, object, and scrambled wh-sentences) as opposed to yes/no questions share the following aspects: 1) encountering *wh*-phrase, 2) checking *wh*-phrase–Q- particle dependency, and 3) semantic specification of the speaker’s intention. If each condition shares similar brain regions, it may be associated with those processes.

We expected the above second and third processing of the *w*h-question comprehension in wh-in-situ language to be in common or compatible with wh-movement language. The *wh*-phrase–Q- particle dependency processing is equivalent to the filler-gap dependency processing for identifying the scope. Thus, brain regions including but not limited to the IFG (or Broca’s area) reported in Ben-Shachar, Palti [9] are of interest. Besides the syntactic processing, from the perspective of cognitive comprehension, differences in the usual process for a *wh*-question compared to that of a yes/no question would be associated with detecting a clue to specifying the intention of the speaker or writer and demanding attention based on the clue. In respect of encountering an interrogative wh-word and thus eliciting attention for resource allocation in the middle of a sentence, we focused on the salience and attention processing, which is generally attributed to the ventral salience and attention system with the anterior insula (aINS) as a core center [11, 12]. The aINS is consistently found to be involved in novel stimuli regardless of the sensory modality and coordinates the dorsal attention system [13] by switching the central-executive network and the default-mode network [14]. The aINS is also involved in complex sequence processing [15] and in speech production with the articulatory network [16]. According to previous literature on the functional role of the aINS, we expected the involvement of the aINS as a base for salience detection leading to prioritization in comprehending *wh*-questions correctly in the *wh*-in-situ languages. This process is expected to work with the IFG, for syntactic processing, such as Q-dependency, and semantic processing, such as prioritization for capturing the speaker’s intention, called covert wh-movement in the LF level in the wh-in-situ languages.

## 2. Methods

### 2.1. Subjects

Twenty-three native Korean adults (12 males and 11 females) participated in the current experiment. Participants’ age ranged from 20 to 27 (mean age, 23.2; standard deviation [s.d], 2.2). All had normal vision, had no neurological disorder, and gave written informed consent before participation. All participants were right-handed, according to the Korean version of Edinburgh Handedness Inventory [17] with handedness score of mean = 89.6, s.d. = 15.13 (range: 53.8–100). They were all paid for their participation. The Internal Review Board (KHSIRB-21-584(RA)) approved this study at Kyunghee University. The recruitment period for this study started on July 25, 2022 and ended November 4, 2022.

### 2.2. Materials

A total of 190 clusters of sentences were constructed (see examples in Table 1). Following Ben-Shachar et al. (2004), we used embedded questions because they permit a straightforward comparison with no-movement questions. We presented Korean-embedded questions of four types: yes/no questions, subject and object *wh*-questions, and scrambled *wh*-questions (See **Table 1**). When constructing sentences, four Korean verbs that take embedded questions as their complements were used: 물었다 (asked), 확인했다 (checked), 알아냈다 (found out), 잊었다 (forgot). The four verbs were selected based on a high frequency/familiarity criterion. Their frequency ranges from 50 to 650 according to National Institute of Korean Language; however, there was no difference in familiarity ratings (on a scale of 1 to 5) among the four verbs (*F* = 0.34; *p* = 0.80). Participants in the familiarity ratings were 25 adults (14 males; average age 27.8 years, range 23–32). They were native speakers of Korean and did not participate in the fMRI experiment. Of note, the verb ’묻다(ask)’ may optionally be accompanied by a dative case particle, a feature that appears to distinguish it from the other three verbs. As we have refrained from incorporating an indirect object in the sentences employing this verb, the apparent differentiation is presumed to have had no discernible impact on sentence comprehension. Each verb is repeated ten times in all conditions. All verbs and embedded questions were in the past tense. For each condition, 40 sentences of different clusters were chosen, such that the mean length of the sentences in each condition was identical (7 words, average size = 19.5 syllables). Thirty nonsense stimuli were included to check if participants were paying attention to the task.

**Table 1:** Conditions and stimulus example. “Nom,” “Acc,” “Top,” and “Q” are abbreviations for a nominative, accusative, topic marker, and a question particle, respectively. In Korean, case particles indicate subject and object when attached to a noun.

Conditions	Example
A: Embedded yes/no questions (nonWH)	나는 그 남자가 그 책을 샀는지 확인했다. I-Top the man-Nom book-Acc bought-Q checked ‘I checked if the man bought a book.’
B: Embedded Object *wh*-questions (objWH)	나는 그 형사가 **어떤** 용의자를 붙잡았는지 물었다. I-Top the detective-Nom which suspect-Acc Caught-Q asked ‘I asked which suspect the detective caught.’
C: Embedded Subject *wh*-questions (subjWH)	나는 **어떤** 관광객이 그 음료를 주문했는지 물었다. I-Top which tourist-Nom the drink-Acc ordered-Q asked ‘I asked which tourist ordered a drink.’
D: Embedded scrambled *wh*-questions (sWH)	나는 **어떤** 주식을 그 투자자가 구매했는지 물었다. I-Top which stock-Acc the investor-Nom purchased-Q asked ‘I asked which stock the investor purchased.’
E: Nonsense	나는 그 작가가 그 창문을 저었는지 확인했다. I-Top the writer-Nom window-Acc stirred-Q checked ‘I checked if the writer stirred the window.’

### 2.3. Procedure, data acquisition, preprocessing, and statistical analysis

Stimuli were delivered visually for 3 s at an interstimulus interval of 3 s with E-prime 2.1 (Psychology Software Tools Inc., Pittsburg, Pennsylvania, USA) in an event-related design sequence, the order of which was predetermined by using optseq2 (http://surfer.nmr.mgh.harvard.edu/optseq); the presentation is randomized and the time in between stimuli can vary. Participants were asked to judge whether the sentence they see makes sense or not and press a button using a left hand for the sentence deemed not meaningful. This button press was intended to evaluate if subjects paid attention to the task. Since we expected the participants would press keys only for the nonsense condition (thus, motor behaviors may contaminate the brain activations for wh-movement), we excluded the nonsense condition from the fMRI analysis. All subjects were given a practice session outside the scanner on a subset of the stimuli to familiarize themselves with the experiment.

Participants were scanned using a 3-T MRI system (Siemens Trio 3 T magnet) with a standard 12-channel “matrix” head coil. fMRI was performed to measure brain activation using the BOLD signal reflected in the T2* weighted continuous echo-planar imaging (EPI) sequence [66 slices; matrix size, 110x110; slice thickness, 2.0 mm without slice gaps; voxel size, 2.0x2.0x2.0 mm^3^; repetition time (TR), 2,500 ms; echo time (TE), 23 ms; flip angle, 80; the field of view (FOV), 220 mm]. Structural T1-weighted anatomical volumes were obtained after two fMRI runs using spoiled gradient recalled echo (sagittal orientation, the thickness of slices, 0.8-mm thick; TR, 2,500 ms; TE, 1.87 ms; FOV, 256 mm). All fMRI images were preprocessed using a conventional preprocessing protocol, including realignment for motion correction, slice timing correction, co-registration to a reference T1-weighted image, spatial normalization to a standard Montreal Neurological Institute (MNI) template, and smoothing with a 6-mm full-width-at-half-maximum (FWHM) Gaussian filter using SPM12 toolbox (http://fil.ion.ucl.ac.uk/spm, Wellcome Department of Cognitive Neurology, London, UK) [18].

After applying a high-pass filter with a cut-off frequency of 128 seconds, the fMRI time series of all the voxels in the template space underwent a general linear model (GLM) analysis with condition-specific timing convolved with a hemodynamic response function as regressors and with the six motion parameters as nuisance regressors, in the individual level using SPM12. A first-level (individual-level) GLM analysis was conducted with contrasts for conditions separately for each subject.

For the first-level analysis, we were concerned about the orthogonality of object *wh*- and subject *wh*-conditions because no significant difference between the two was found in the previous literature [9]. If the brain activations between subject *wh*- and object *wh*- conditions in the second-level analysis do not differ at the linguistic brain regions, it implies that the two conditions recruit almost the same linguistic processes. In that case, we would merge the two regressors to a regressor of *wh*-condition to improve the statistical power by reducing the number of model parameters and making condition-specific signals orthogonal.

At the random-effect level between subjects, the parameter estimates for each condition in the first-level analysis were used in the flexible design to conduct repeated measures analysis of variance (ANOVA) using SPM12. The contrasts of interest in the second-level analysis included: [*wh*-question without scrambling–yes/no question (gWH–nonWH)], [*wh*-question with scrambling–yes/no question (sWH–nonWH)], [*wh*-question regardless of scrambling–yes/no question (WH–nonWH)], and [*wh*-question with scrambling–*wh*-question without scrambling (sWH–gWH)]. We used for WH-nonWH a contrast vector [1/3 1/3 1/3–1] for the objWH, subjWH, sWH, and nonWH.

For this group-level analysis, we set a cluster-level criterion with a voxel-level threshold *p* < 0.005 and the extent threshold *k* > 200 voxels and satisfying topological false discovery rate (FDR) < 0.05 (Chumbley, Worsley [19]) corrected by cluster-level for multiple comparisons. As a posthoc analysis, the percent signal changes for significantly detected clusters were calculated by counting three mm-diameter sphere regions around the cluster’s peak using MarsBaR software [20]. This posthoc analysis was not intended to generate new statistical inferences or bolster our current findings. Rather, its primary purpose was to succinctly summarize the activation patterns observed across all conditions.

## 3. Results

As a behavioral test for the participant’s involvement, the nonsense or meaningfulness judgment’s accuracy was, on average, 90.6 percent (standard deviation: 4.24), showing strong participation in the task with relative easiness. As a test for the difference between subject and object *wh*-questions, we found no significant differences in brain activation in the conventional language areas except for the bilateral fusiform gyrus (posterior part) (**Table 2**). Thus, the subsequent analysis was based on the first-level analysis by merging object and subject *wh*-conditions as a general *wh*-condition.

**Table 2:** Brain activation results. gWH: a condition of the general sentence with wh-element. sWH: a condition of the scrambled sentence with wh-element. WH: a condition of the sentence with wh-element (both gWH and sWH). nonWH: a condition of the sentence without wh-element.

Region	Coordinate x,y,z	Zmax	Cluster size	Region	Coordinate x,y,z	Zmax	Cluster size
*WH> nonWH*				*sWH > gWH*			
L superior frontal gyrus (BA6)	-16, 4,60	4	377	R/L supplementary motor area (BA32)	14,22,44	3.69	209*
L supplementary motor area (BA6)	-4,16,54	3.71	-	R/L superior frontal gyrus (BA8)	4,28,54	3.49	-
R supplementary motor area (BA32)	14,20,48	3.48	-	*sWH < gWH*			
L anterior insula (BA48)	-32,12, 8	3.96	681	R superior parietal lobule (BA3)	28,-36,50	4.06	600
L frontal operculum (BA47)	-40,22, 4	3.91	-	R postcentral gyrus/precuneus (BA3)	18,-40,72	3.98	-
R frontal operculum (BA48)	34,18,10	3.93	385	L precuneus (BA5)	-6,-44,60	3.69	722
R caudate (BA48)	22,18,14	3.78	-	L superior parietal lobule (BA7)	-22,-52,54	3.63	-
R calcarine cortex/cuneus (BA19)	24,-60, 6	3.71	252				
L calcarine cortex/cuneus (BA17)	-2,-66, 6	3.52	239				
*gWH>nonWH*				*sWH>nonWH*			
L superior frontal gyrus (BA6)	-16, 4,60	4.44	316	R supplementary motor area (BA32)	10,20,48	4.03	454
L supplementary motor area (BA6)	-2, 8,54	3.73	-	R medial superior frontal gyrus (BA8)	6,30,44	3.74	-
L anterior insula (BA48)	-36, 8, 8	4.35	494	R frontal operculum (BA48)	34,18,12	3.82	302
L frontal operculum (BA47)	-40,24, 4	4.08	-	R caudate	18,20,18	3.54	-
R calcarine cortex (BA18)	14,-76, 8	4.14	1514	L anterior insula (BA48)	-30,18, 8	3.61	480
L calcarine cortex/cuneus (BA17)	-6, -74,14	3.81	-	L frontal operculum (BA47)	-42,22, 2	3.6	-
L middle/superior temporal lobe (BA37)	-60, -58, 8	4.01	534	L anterior insula/frontal operculum (BA48)	-40,14,-2	3.5	-
L supramarginal gyrus (BA42)	-62,-42,18	3.74	-	*Subject WH > Object WH* ^*†*^			
L angular gyrus (BA7)	-30,-60,52	3.88	222	L occipital fusiform gyrus/inferior occipital gyrus (BA18)	-18,-92,-6	3.91	572
L superior parietal lobule (BA40)	-32,-54,58	3.45	-	R occipital fusiform gyrus/inferior occipital gyrus (BA18)	16,-80,-8	3.67	281
R anterior insula/caudate (BA48)	22,18,14	3.73	231				
R frontal operculum (BA48)	34,18,10	3.6	-				
R postcentral gyrus (BA2)	16,-42,64	3.65	398				
R superior parietal lobule (BA40)	36,-48,56	3.6	-				

*p* < 0.005, cluster FDR > 0.5 or cluster size > 200, x, y, z: Montreal Neurological Institute coordinates in mm, Zmax: maximum Z within a cluster, L: Left R: Right. “-” in the cluster size indicates that this coordinate is a peak location that belongs to the cluster listed immediately above.

* does not meet FDR<0.05.

^†^ is evaluated by contrasting regressors for subject and object wh-questions.

**Fig 1:**
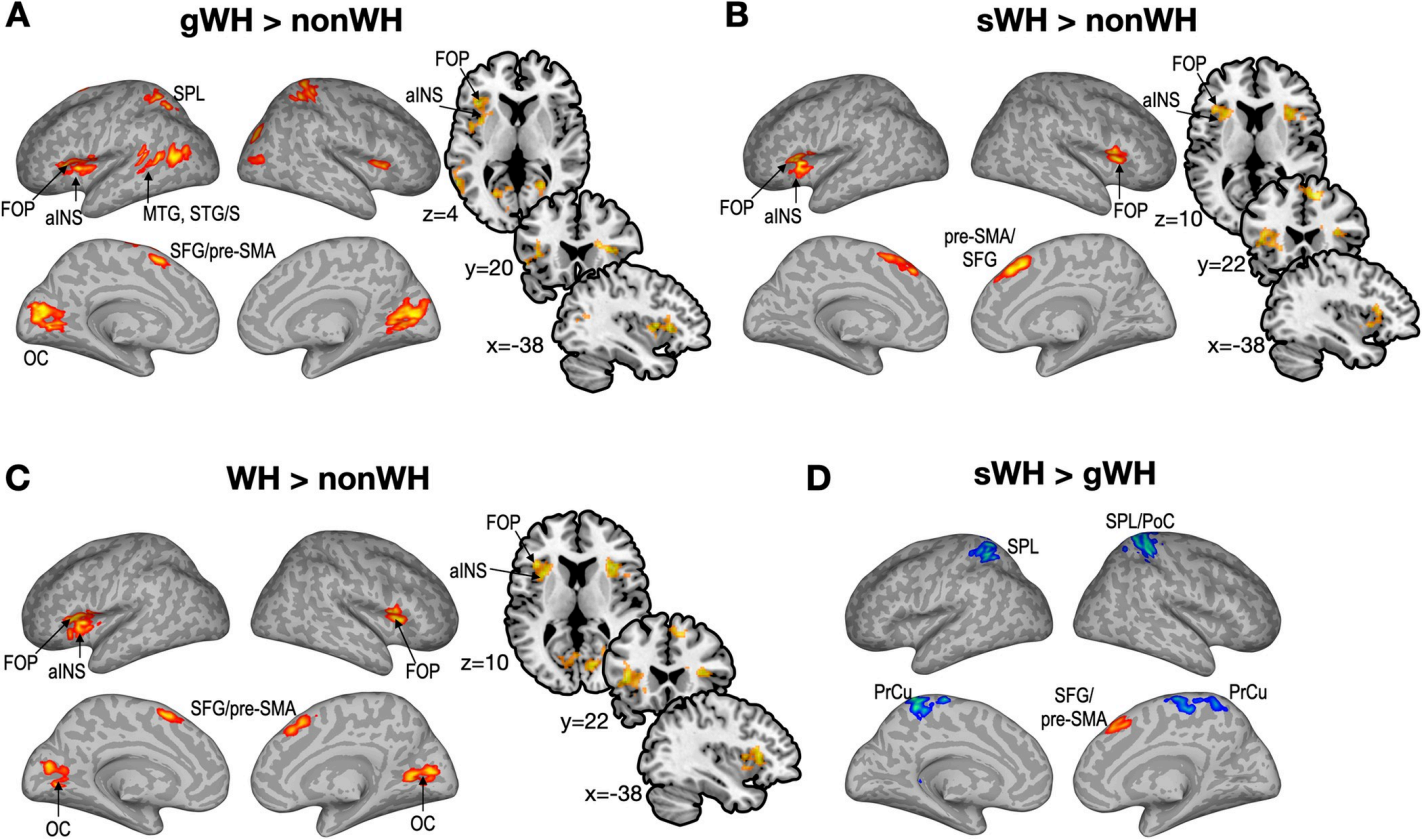
Statistical parametric maps for the contrasts. A. Statistical results for the general *wh*-question without scrambling condition (gWH) versus the yes/no question condition (nonWH) are displayed over the cortical surface and section views. B. Statistical results for the scrambled *wh*-question condition (sWH) versus the nonWH condition are shown. C. Statistical results of WH conditions (general and scrambled WH) versus nonWH conditions are displayed with red colors (increased activation in the WH conditions compared to the nonWH condition). D. Results of the scrambled WH versus the general WH conditions are shown with red colors and blue colors indicating increased and decreased activation, respectively, for the scrambled WH condition compared to the general WH condition. The clusters with a threshold *p* < 0.005 and cluster size *k* > 200 are displayed (all clusters are FDR < 0.05, except for SFG/pre-SMA in D, see Table 1). aINS: anterior insula, FOP: frontal operculum, SFG: superior frontal gyrus, pre-SMA: pre-supplementary motor area. OC: occipital cortex (calcarine and cuneus), SPL: superior parietal lobule, PrCu: precuneus, PoC: postcentral cortex, MTG: middle temporal gyrus, STG/S: superior temporal gyrus and sulcus. BA: Brodmann area. x, y, and z indicate MNI coordinate.

**Fig 1** and **Table 2** summarize the statistical results for contrasts between conditions. **Fig 1A** shows the increased activation for the general wh-condition without scrambling (gWH) than the baseline condition, i.e., yes/no question without *wh*-element (nonWH). The activity was present bilaterally in the aINS, FOP, superior parietal lobule (SPL), and occipital cortex (OC), as well as in the left supramarginal and angular gyri, middle and superior temporal gyri and sulci, and pre-supplementary motor area (SMA). **Fig 1B** shows the activation result for the contrast of *wh*-condition with scrambling (sWH) versus yes/no question. Significant activations were found in the bilateral frontal operculum, left anterior insula, right pre-SMA, and superior frontal gyrus (SFG). Compared to the baseline condition, *wh*-stimuli, regardless of scrambling, induced more significant activations bilaterally in the FOP, pre-SMA and calcarine/cuneus, and the left aINS (**Fig 1C**). No significant differences were found in the vice-versa contrast. Compared to the general *wh*-question, the scrambled *wh*-question condition (sWH) had increased activity in the right SFG/pre-SMA while decreased activity in the bilateral precuneus and superior parietal lobule (**Fig 1D**).

**Fig 2:**
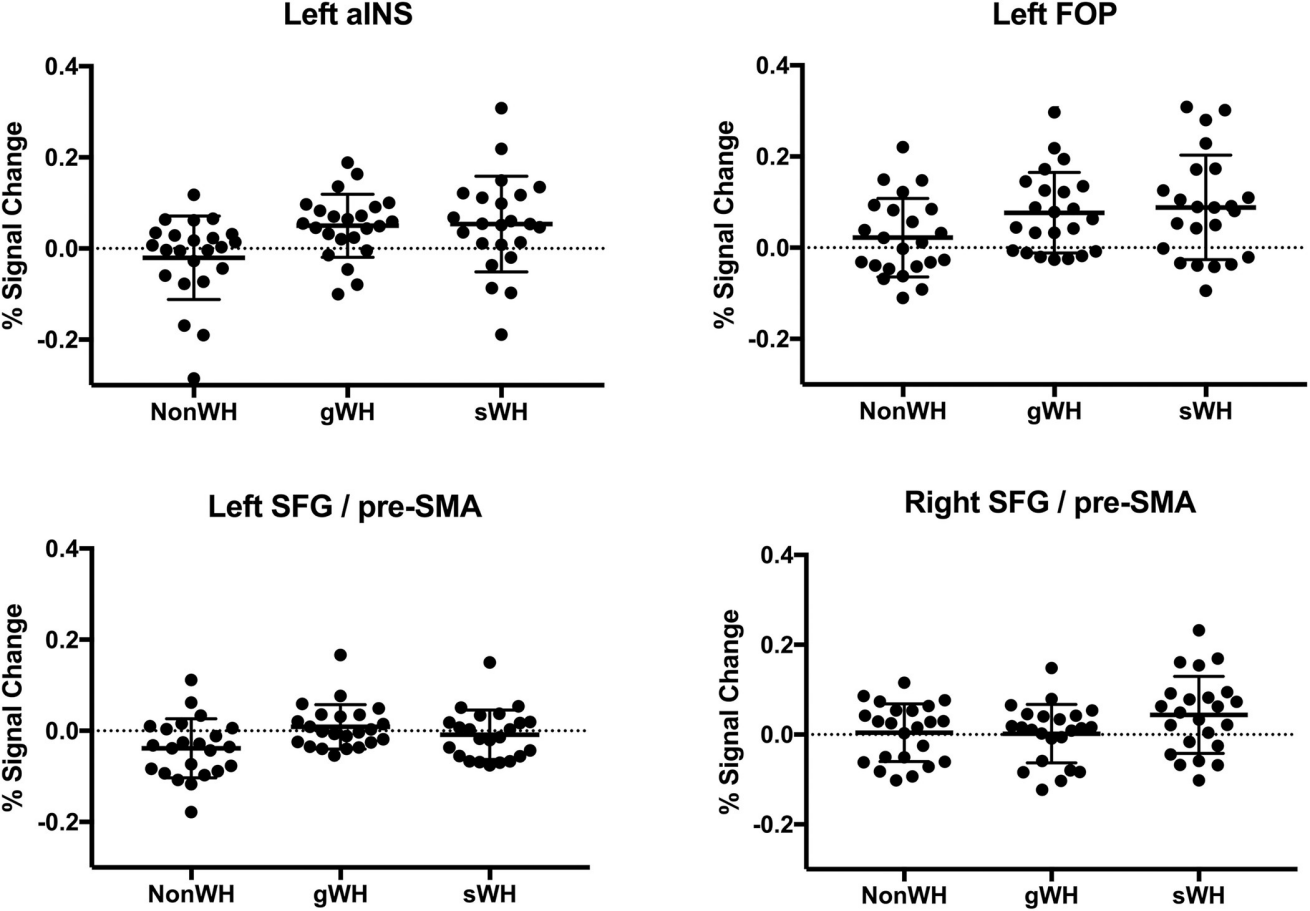
Percent signal changes. Percent signal changes (mean and standard error) are displayed for the yes/no question (non-WH), the general wh-question (gWH), and the scrambled wh-question (sWH) at the left anterior insula (aINS;-32/12/8), left frontal operculum (FOP;-40/22/4), left superior frontal gyrus (SFG) and posterior part of the pre-supplementary motor area (pre-SMA) (-16/4/60) and the right SFG and anterior part of the pre-SMA (14/22/44).

**Fig 2** summarizes percent signal changes for the yes/no question (non-WH), the general wh-question (gWH), and the scrambled wh-question (sWH) at the local maxima of the left anterior insula (aINS;-32/12/8), the left frontal operculum (FOP;-40/22/4), the left superior frontal gyrus (SFG) and posterior part of the pre-supplementary motor area (pre-SMA) (-16/4/60) and the right SFG and anterior part of the pre-SMA (14/22/44). The aINS shows a higher percent signal change in gWH than non-WH and a higher percent signal change in sWH than non-WH. The left FOP shows condition differences with a higher percent signal change in gWH and sWH than non-WH. The left SFG/pre-SMA has a higher percent signal change in gWH than sWH and non-WH and a lower percent signal change in sWH than non-WH. The right SFG/pre-SMA shows a percent signal difference with a higher percent change in sWH than gWH.

## 4. Discussion

In the current study, we used fMRI to explore brain responses involved in comprehending *wh*-question sentences in Korean, a *wh*-in-situ language. We found increased involvement of the left aINS, bilateral FOP, bilateral SFG, and bilateral occipital cortex in the *wh*-condition versus the yes/no condition. Among these regions, in line with a previous study [9], we primarily focused on the aINS and the FOP, which was partly expected for processing *wh*-questions with interrogative wh-element in the middle of sentences.

As there is no apparent *wh*-movement in sentence structure in Korean wh-questions, we anticipated that if we found the same engagement of the IFG (Broca’s area, specifically, Brodmann areas (BA) 44 or 45) as the overt *wh*-movement in Hebrew [9], the IFG involvement would relate to general syntactic processing abilities regardless of the existence of overt *wh*-movement, rather than the processing specific to the overt *wh*-movement observed in the study of Hebrew [9].

However, increased activity for *wh*-in-situ questions was not found at the IFG subregions but at the FOP in the online comprehension of *wh*-element in Korean. According to recent cytoarchitectonic analyses, the FOP (BA 47 or BA 48) is considered a structural correlate of Broca’s region [21] and is connected with and often undifferentiated from the IFG [22]. In terms of the interconnected areas of the temporal and frontal lobes known as the language center [23], the FOP plays a critical role in early language processing, as it is known to be phylogenetically older than the IFG and BA 44 in particular [24]. Several neuropsychological studies have linked lesions in the FOP to Broca’s aphasia [25–27]. The FOP’s involvement has been shown in online syntactic structure-building processes, syntactic violation [28], and other syntactic information [29, 30]. An increase in sequence complexity induces increased activity in the FOP as well as the aINS [31]. These linguistic processes may be associated with the FOP’s broad role as a network core [32, 33] controlling other brain regions for diverse cognitive tasks, for example, in top-down selective attentional modulation [34].

The aINS and FOP are part of the ventral salience and attention network [11, 12, 35] which, together with the dorsal anterior cingulate cortex [36], play a role in salient processing [37, 38] regardless of sensory modality. The aINS coordinates the attention-related salience system during task performance [32, 39, 40]. In linguistic tasks, the aINS, in combination with the FOP, contributes to coordinating and planning speech articulations [41]. Bohland and Guenther [15] have argued that the sensitivity to complexity shown by the aINS and FOP may be involved in syllable sequence representation as an integration site of lower- and higher-level representations of speech. Woolnough, Forseth [42] noted that the FOP had not been considered separately from the aINS in fMRI studies of speech production and perception, which may be attributable to technical issues such as low spatial resolution and smoothing difficulties. Using intracranial electroencephalography, the authors found that speech-related activities occurred mostly in the FOP rather than the aINS. In particular, the timing of activity suggested that the FOP holds a preparatory role previously believed to be held by the aINS [42]. To sum up, the aINS may be more associated with bottom-up perceptual processing of a sentence (such as salience detection), while the FOP may be more related to the coordination of sequence processing with top-down selective attention [34].

Considering the involvement of the aINS in diverse cognitive and linguistic tasks listed above, we attribute increased activation in the aINS to the online and ongoing process of a salient interrogative word, i.e., the *wh*-element. When unexpectedly encountered in the middle of a sentence in a *wh*-in-situ language (the position of a *wh*-element in a sentence is not predetermined), a *wh*-element is a salient interrogative word. It may induce arousal for attention to comprehend all the words in the sentence, a task for which the aINS may be more responsible. Comprehension of *wh*-questions may not be confined to salient perception of the interrogative word but may extend to subsequent cognitive and linguistic processing. For the interrogative word encountered, the brain may allocate attention to search the scope while holding the previous part of the sentence, prioritizes the words of the sentence, and specifies the question of interest for the speaker, all of which may be attributable to the FOP.

This early part of *wh*-question processing may not typically be needed in English-like languages, where the most important word, the *wh*-element, has already been placed in the first place in the overt syntax—this is called “*wh*-movement” from the speaker’s perspective. As it is a crucial word and its priority was already determined by the speaker, the perceiver may then focus more on analyzing the sentence based on the type of *wh*-element (e.g., “what,” “who”). This may explain why Ben-Shachar, Palti [9] did not detect a significantly increased involvement of the aINS in the assessment of *wh*-movement versus no movement (yes/no question) in Hebrew.

Given the IFG’s engagement in processing sentences with overt *wh*-movement [9], it is plausible that the FOP, considered to represent an evolutionarily older system than the IFG in the realm of grammar processing [24, 43], plays a role in later stages of syntactic or semantic processing influenced by the aINS. In Korean, this involvement could encompass the reordering of *wh*-elements to initiate the subordinate clause at the LF level. Given its proximity, we liken the FOP’s function to that of the IFG. From this perspective, we posit that the IFG’s activity during *wh*-question comprehension in Hebrew, a language employing overt *wh*-movement [9], might pertain to syntactic operations such as determining scope using the *wh*-dependent property (or "gap") within the comprehension process for *wh*-questions. This inference arises from the similarity in brain activation observed across the subject and object *wh*-question contrast as well as the scrambled and general *wh*-question contrast—indicating consistent neural responses in comprehension, irrespective of position (subject vs. object *wh*-sentence, i.e., differently positioned *wh*-phrase) or movement (scrambled vs. general *wh*-sentence).

In addition to the aINS and FOP, we also found involvement of the left SFG and pre-supplementary motor area (SMA) in the perception of *wh*-questions; these areas are known to be part of or to work in conjunction with the salience or attention system. Grewe, Bornkessel [44] reported the involvement of the FOP and pre-SMA in object-first versus subject-first sentence comprehension, which is consistent with our finding that these regions were active during the processing of non-canonical (salient) word order sequences. Our finding of greater involvement of the primary visual cortex for the *wh*-question relative to the yes/no question condition is not surprising. More attention and time may be required to decide whether the *wh*-question makes sense at the syntactic and semantic levels. The occipital lobe showed increased activation for attention-arousing stimuli [45], similar to a *wh*-interrogative sentence.

We cannot rule out that the pre-SMA identified in our study might correspond to the Supplementary Eye Field (SEF), known for its broad role in brain function, including saccadic eye movement coordination, complex decision-making, motor planning integration, rule-based action encoding, outcome evaluation, and performance monitoring [46–52]. The comprehensive role of the SEF could intersect with cognitive processes in comprehending wh-questions, compared to yes/no questions. Given the fixed presentation time (~3 sec) across conditions, a relatively low gaze angle and ease of processing sentence stimuli in our study, and no significant activation at the frontal eye field or posterior parietal lobule for overt eye movement, the effect associated with eye movement may be subtle or potentially covert, highlighting the need for further research to define the precise role of the pre-SMA in these cognitive processes.

The subject-object contrast in *wh*-questions was not significant in conventional language areas. Subject and object *wh*-questions are argued not to involve syntactic movement in *wh*-in-situ languages. Since we found no significant difference in language regions between the two constructions, it is not the syntactic position of *wh*-element that matters. This is consistent with previous reports that there was no activation difference between the two conditions in overt *wh*-movement languages [9], where subject and object *wh*-elements occur in the first place with high certainty or almost as a rule. This suggests that the position of the *wh*-element itself does not contribute highly to the current inferior frontal lobe activation in *wh*-question processing. Instead, perceiving the critical (salient) word in its unfixed position is vital in recruiting the aINS or FOP.

The scrambled *wh*-condition versus yes/no condition also revealed increased activation in the FOP and aINS. However, the scrambled *wh*-condition did not increase activity in the FOP and aINS regions more than the general *wh*-condition. This finding was somewhat unexpected since we had anticipated that the comprehension of scrambled *wh*-questions, which deviated from the canonical syntactic form but contained the speaker’s emphasis, is more costly than canonical sentences, as found in Japanese and other languages [53].

The absence of increased IFG activation for scrambled wh-sentences compared to general wh-sentences in our study may be due to our specific task design and the use of the [+wh] feature in scrambled objects. While studies like Bornkessel et al. [54] and Grewe et al. [55] indicate that the IFG activation varies with thematic role hierarchy and animacy structures, their focus on object non-wh noun phrases and/or passive voice contrasts with our emphasis on object wh-phrases in active voice. In a similar vein, Kim et al. [56] and Ohta et al. [57] noted increased IFG activation in scrambled sentences, but their methodologies differ significantly from ours in terms of clause type (main vs. embedded), the utilization of [+wh] feature, and the specific task design. In addition to these distinctions, because for both scrambled wh- and general wh-questions, the comprehenders directed a significant amount of their attention or syntactic demands towards wh-phrases, we posited that the scrambling effect (also referred to as the syntactic loads effect) might have been diminished, potentially leading to an absence of significant activation differences for the scrambled contrast in the aINS/FOP (or IFG).

Meanwhile, increased activation for the scrambled sentence was shown in the right anterior part of the supplementary area (pre-SMA), a dorsal salient network, with mostly decreased activation in the superior parietal lobe and precuneus **(Fig 1D)**. Although we cannot straightforwardly interpret this finding, we speculate that the scrambled *wh*-question is more unusual and salient than the general *wh*-question, as the location of the *wh*-word is unexpectedly changed from the canonical *wh*-question sentence. This requires redistribution of comprehensive brain resources to attend to the processes specific to scrambled *wh*-sentences, requiring more cognitive processing to judge meaningfulness than general *wh*-questions. The scrambled *wh*-question includes a stronger intention of the speaker; that is, the speaker intentionally scrambles the sentence to focus more on the scrambled *wh*-word. Thus, increased activation in the pre-SMA and decreased activation in other areas may be associated with a complex inference process to determine whether the sentence makes sense or to interpret the speaker’s intention through non-canonical word order. The complex processing involved in comprehending scrambled wh-sentences might necessitate a reallocation of brain resources towards more focused processing of these salient stimuli. As previously discussed, there is a possibility that these cognitive efforts are related to the faculty of the SEF if the significantly different region in the pre-SMA is part of the SEF. However, these ideas are speculative and warrant further research to validate the specific functions of these brain areas. Additionally, a comprehensive study is needed to understand how the brain differentially engages in the comprehension of scrambled wh-questions compared to general wh-questions.

The current study has several limitations. We did not assess salience scores, which would specify the current functional activity. Besides, the collection of behavioral data, specifically response times associated with each item, was deliberately avoided. This decision was motivated by the intention to mitigate potential interference with motor-related cerebral regions implicated in the process of syntactic analysis. It is noteworthy that the benefits of acquiring response time data through button-press responses are counterbalanced by the attendant drawbacks, chief among which is the inability to definitively discern whether the observed activation of motor-related regions is attributable to syntactic processing or the act of button-pressing itself [58, 59]. As we asked participants whether each sentence is sensible or not, we expected the complexity difference in determining meaningfulness/sensibleness between the yes/no and wh-questions, potentially reflected in the response time and thus brain activation, would be minimal.

It would be intriguing to determine whether the current results can also be observed in other *wh*-in-situ languages, which would suggest the universality of *wh*-in-situ processing. Direct comparison with bilingual participants for Korean and English will be further researched to identify the common and language-specific syntactic processing in the comprehension of wh-questions.

In summary, based on the results of our functional MRI study of *wh*-question comprehension, we argue that covert *wh*-movement in the LF level reported in the previous literature [2–6] may be associated with syntactic and semantic salience, syntactic processing for wh-dependency and prioritization by moving the crucial words to the beginning of a sentence in the semantic interpretation level. For this process, a *wh*-element functions as a salient stimulus for the listener or reader to immediately capture what the speaker intended. We speculate that the aINS and FOP work together to process salient sentences and coordinate (or prioritize) the pieces of meaning in an orderly manner.

## Supporting information

S1 Appendix (DOCX)
