# Supplementary material for: Involvement of the anterior insula and frontal operculum during wh-question comprehension of wh-in-situ Korean language
Source: PLoS One. 2024 Apr 26;19(4):e0298740. doi: 10.1371/journal.pone.0298740 (PMC11051625; doi:10.1371/journal.pone.0298740)
Supplement: S1 Appendix — (DOCX) [file pone.0298740.s001.docx]

**Appendix 1**

**Example 1**: wh-in-situ question.

The following is an example of a wh-question in Korean.

1. ne-nun mwues-ul mek-ess-ni?

You-Top what-Acc eat-Past-Q

‘what did you eat?’

The LF version of the above sentence is shown below:

1. mwues-ul_1_ [ne-nun t_1_ mek-ess-ni]

**Example 2**: wh-dependency in Korean.

In Korean wh-questions, wh-dependency exists, as shown in the following example.

a. Embedded clause wh-question John-un [Mary-ka **mwues-ul** mek-ess-**nunci**] mwul-ess-**ni**? John-TOP Mary-NOM what-ACC eat-PST-Q ask-PST-Q ‘Did John ask what Mary bought?’ b. Matrix clause wh-question John-un [Mary-ka **mwues-ul** mek-ess-ta-ko] malha-yess-**ni**? John-TOP Mary-NOM what-ACC eat-PST-DECL-that say-PST-Q ‘What did John say Mary ate?’

In both cases, the wh-phrase is in the embedded clause, but the position of the Q-particle differs. In (a), the Q-particle is in the embedded as well as the main clause while in (b), it is only in the main clause. This distinction also results in the difference in the interrogative scope. In (a), the Q-particle in the embedded clause binds the interpretation of the wh-phrase in the same clause, functioning as a scope barrier for the wh-phrase. In (b), however, the wh-phrase has a wide-scope reading, as shown from the interpretation. Thus, the Q-particle’s position plays a central role in determining the scope of a wh-interrogative sentence. Unlike in English, where a wh-element is displaced to the left of its gap and marks its own scope, in Korean or Japanese, a Q-particle appears to the right of the corresponding wh-phrase and marks its scope.

**Example 3**: scrambled wh-question in Korean.

In the following example, the *wh*-object phrase has been scrambled out of its normal position into the position before the subject:

1. etten umlyo-lul_1_ [ku-ka t_1_ cwumwunha-yss-ni]? which drink-Acc he-Nom order-Past-Q ‘Which drink did he order?’
